# Should we maintain baby hatches in our society?

**DOI:** 10.1186/1472-6939-14-9

**Published:** 2013-02-22

**Authors:** Atsushi Asai, Hiroko Ishimoto

**Affiliations:** 1 Department of Bioethics, Kumamoto University Graduate School of Medical Science, Kumamoto University, Kumamoto, 1-1-1 Honjo, Kumamoto 860-8556, Japan; 2 Department of Bioethics, Kumamoto University Graduate School of Life Science, Kumamoto University, 1-1-1 Honjo, Kumamoto 860-8556, Japan

**Keywords:** Baby hatch, “Stork’s Cradle (Kounotori no Yurikago)”, Babyklappes, Japan, Child abuse, Child abandonment, Abortion

## Abstract

**Background:**

A baby hatch called the “Stork’s Cradle” has been in place at Jikei Hospital in Kumamoto City, Japan, since May 10, 2007. Babyklappes were first established in Germany in 2000, and there are currently more than 90 locations. Attitudes regarding baby hatches are divided in Japan and neither opinions for nor against baby hatches have thus far been overwhelming. To consider the appropriateness of baby hatches, we present and examine the validity of each major objection to establishing baby hatches.

**Discussion:**

There are various objections to baby hatches as follows: It violates a child’s right to know the identity of his or her biological parents by allowing anonymous birth; it neglects fulfillment of the biological parents’ basic obligation to raise their child and its very availability induces abandonment of infants; some people abuse it for very selfish reasons; it cannot save babies’ lives; the rights of one parent can be ignored if the other surrenders a child without his or her consent; it puts a baby in medical jeopardy; and it has no clear legal basis. The authors would argue that there are many plausible refutations for each objection mainly based on priority of child’s right to life, pregnant women’s vulnerability and necessity of anonymity, social responsibility to protect and raise children, differences between dropping a child off at a baby hatch and child neglect, limited function of social childcare center, inevitability of abuse by a minority of people, necessary distinction between outcomes that occur only because baby hatches exist and those that occur regardless of their existence, important local direct and upmost measures for women in trouble, and difference between ambiguous legality and illegality.

**Summary:**

We argue that a certain number of baby hatches should continue to be established as a last resort, in a form that can maintain anonymity if the parent dropping the child off so desires. It should be supported if it is initiated with good intentions; if the maximum possible effort is made at said facility to protect the interests, rights, and safety of the child; and if no clear evidence of harm exists.

## Background

### Current situation regarding baby hatches in general and the “Stork’s Cradle (Kounotori no Yurikago)” of Kumamoto City

A baby hatch called the “Stork’s Cradle” has been in place at Jikei Hospital in Kumamoto City, Japan, since May 10, 2007. In the five years since its establishment, 81 children have been placed in its care. Eight of the 40 male and 41 female children were disabled. Since 2002, Jikei Hospital has taken measures to protect the lives of fetuses and children, for example, by conducting telephone counseling for women worried about pregnancy. The hospital established a facility that accepts children anonymously, modeled on German *Babyklappes,* in hopes of saving newborn babies that die from abandonment as well as lives lost to abortion [1]. Babyklappes were first established in Germany in 2000, and there are currently more than 90 locations. Facilities with similar functions exist in 20 countries, including Poland, the Czech Republic, Hungary, Italy, Austria, Vatican City, USA, India, and South Africa [2,3].

Thirty parents who placed their babies in the care of the “Stork’s Cradle” within the past two years provided the following reasons for doing so: poverty (nine responses), unmarried (nine responses), public image/unwillingness to enter in family register (six responses), problems with partner (six responses), and extramarital affair (four responses). A report acknowledged that many children had been saved by the “Cradle.” On the other hand, the report also noted cases in which children were dropped off for simplistic reasons such as, “can’t find a facility to look after the child while I work” and “can’t bring up the child because I will study overseas,” and called for measures that would encourage parents to seek counseling in advance [1,4].

Attitudes regarding baby hatches are divided in Japan. Baby hatches are often criticized as evil and encouraging irresponsible child abandonment; moreover, people are not necessarily accepting of this experimental effort [5]. In a July 2007 Kumamoto Nichinichi Newspaper survey of eligible voters in Kumamoto Prefecture, 49% responded that “the Cradle is necessary,” 14.9% responded that it was “unnecessary,” and 33% responded that they “could not say.” The fact that many people were unable to decide demonstrates that the existence of the “Cradle” is complicated [6]. In a 2007 Asahi Shimbun online survey of 4400 people, approximately 59%, 28%, and 13% of respondents replied, “(baby hatches are) necessary,” “(baby hatches are) unnecessary,” and “not sure,” respectively, and an online poll of 1092 people by the Mainichi Shimbun found that 63% were in favor of and 37% were against baby hatches [7,8].

Corresponding numbers for and against hatches were found among neonatologists: 54.4% of 68 respondents were for and 38.2% were against baby hatches. Examined separately by age and sex, young female physicians with less than ten years’ experience showed the most support, with 66.7% for and 22.3% against baby hatches. Supporting opinions ranged from “I value it as a life-saving measure” to “It should be implemented carefully given the risk of encouraging child abandonment” [2]. The situation in which there are arguments both for and against baby hatches seems similar in Germany which has the most baby hatches in the world, according to the interviews with scores of people conducted by the Kumamoto Nichinichi Newspaper staff, although the same kind of data based on research surveys or public-opinion poll has not been available [6,9].

In both Japanese and German surveys, neither opinions for nor against baby hatches have thus far been overwhelming. Although the results depend on how the questions are worded, the respondents are either evenly divided or those in favor of baby hatches slightly exceed those against, although many are also undecided. Some view them as necessary despite being opposed to them, while others find them difficult to accept based on personal beliefs but feel they are unavoidable when considering reality [2,3,6-12]. The “Stork’s Cradle” in Kumamoto City and the Babyklappes in Germany continue to survive amidst these circumstances.

To consider the appropriateness of baby hatches, in this article we present and examine the validity of each major objection to establishing baby hatches. Then, we argue that a certain number of baby hatches should continue to be established, literally as a last resort, in a form that can maintain anonymity if the parent dropping the child off so desires. Finally, we conclude that a baby hatch should be supported if it is initiated with good intentions; if the maximum possible effort is made at said facility to protect the interests, rights, and safety of the child; and if no clear evidence of harm exists.

## Discussion

### Critical appraisal regarding major objections to the establishment of baby hatches

#### The baby hatch violates a child’s right to know the identity of his or her biological parents by allowing anonymous birth

This objection holds that a child’s right to know their parents and origins is disregarded for children dropped off at a baby hatch. Article 7 of the 1989 UN Convention on the Rights of the Child declares that “the child shall be registered immediately after birth and shall have the right from birth to a name, the right to acquire a nationality and, as far as possible, the right to know and be cared for by his or her parents” [13]. Discussions of the right of children born from artificial insemination by sperm donation to know their origins have articulated three disadvantages for a child who does not know his or her origins: [1] the lack of genetic information may infringe on the child’s right to health, [2] the inability to exclude the possibility of a consanguineous marriage when the child marries, and [3] the child will lack information on his or her biological parents and his or her own birth, which are crucial to the establishment of independence and identity [14]. These perspectives are all important and undoubtedly must be respected in principle to protect children who are placed in a vulnerable position.

Recently, some countries have started requiring a donor to allow disclosure of his identity if a child born by artificial insemination requests it [14]. In this case, those who do not desire disclosure of their identity are unlikely to become donors. Similarly, parents who wish to remain anonymous and women who wish to conceal their pregnancies may choose abortion, infanticide, or child abandonment if anonymous drop-offs are not allowed. Fixating exclusively on respecting the right of the child to know his or her parents without considering the circumstances will lead to violation of the right to life. If the child cannot survive, then the right to know his or her origins can neither be claimed nor protected. Discussions should thus consider the temporal order of realizing rights. Sakamoto argues that baby hatches give children the right to life, and for newborns, survival is more important than the right to know and be cared for by their parents [15].

Some claim that parents who insist on anonymity when leaving a child at a baby hatch are selfish and irresponsible. In many cases, however, these parents are women who are desperate and lonely with no one to talk to, and perhaps are only able to speak out in anonymity. It is also possible that these parents are isolated and without friends they can talk to intimately or adults they can trust, are unaware of organizations to consult because interpersonal relationships in their lives are limited, or they harbor distrust or hatred of such organizations even if they are aware of them [5]. Some of these parents may be unable to talk using their real names because they fear being discriminated against and criticized as “a selfish and irresponsible human being” [5]. It can be difficult for confused parents to casually approach our country’s social welfare system for consultation [6]. It may be said that it is hard to live as a single mother but easy for her to have an abortion in Japan [16]. Ultimately, they have no choice but to rely on baby hatches. Thus, baby hatches have the societal function of serving as a last resort.

Furthermore, we wonder whether a person may, on occasion, be unable to talk to anyone in truly tough times. Even if the person knows that the listener will be kind and welcoming with a receptive attitude, taking the bold step of consulting with someone is difficult. Consulting with someone is a major decision, and outwardly expressing one’s suffering to a stranger is no easy task. There are probably some women who, embracing their despair alone without being able to confess their troubles to anyone, at some point cross the line and tragically end their lives, together with their unborn or newborn child [6]. Baby hatches provide an alternative for these women.

Therefore, even anonymous use of baby hatches should be allowed. Not all parents should be forced to disclose their identity. In principle, the right of a child to know his or her origins should be higher in priority than the parent’s right to anonymity, based on the vulnerability of children and the importance of the outcome; however, in exceptional cases where the parent is mentally and economically desperate, and the conditions are such that abortion, infanticide, or child abandonment may occur unless privacy is protected, the parent’s right to anonymity should be upheld to protect both the child and parent.

#### The baby hatch neglects fulfillment of the biological parents’ basic obligation of to raise their child and the very availability of the baby hatch induces abandonment of infants

Some object to baby hatches on the grounds that society is approving irresponsible child abandonment on the part of biological parents; the existence of baby hatches will create a demand for child abandonment that did not previously exist and parents will make a hasty and mistaken decision to drop the child off at a baby hatch rather than a public institution [1-3,5,6,17]. In other words, if baby hatches do not exist, parents will try more desperately to raise the child, even in cases of poverty, and will seek assistance from a community facility suitable for dropping off the newborn child in situations where raising the child seems impossible. Taking the “Stork’s Cradle” of Kumamoto as an example, this objection claims that the 81 children placed in its care during a five-year period were “thrown away” precisely because the “Stork’s Cradle” exists.

The basis for this objection is the notion that parents have an unambiguous duty to raise their children, even under desperate circumstances. Furthermore, the perception that baby hatches undermine the heretofore cherished basic ethical values of traditional societies is probably inevitable [6]. To begin with, child rearing by parents or parent-like caregivers has been considered a self-evident fact in modern societies as well as a prerequisite for those societies [5]. Supposedly, baby hatches are inappropriate as social childcare organizations because of their anonymity as well as their short-sightedness and ease of use. From this perspective, if the parents cannot raise the child themselves for some pressing reason, then they should follow the procedures determined by the official child consultation center of the community, and continuously consult with as well as receive assistance from the center.

In fact, when Jikei Hospital began the “Stork’s Cradle” in 2007, the Japanese prime minister expressed strong concerns: “If you bear a child, it is crucial that you take responsibility as a parent; there are facilities to deal with such children, and I feel very uncomfortable about making a facility that allows you to leave a child anonymously”; “I feel it is unacceptable for a father and mother to abandon a child anonymously”; “(The Cradle) ought not to exist” [6]. In addition, there are concerns that abandoning the child at a baby hatch does not truly solve the problem that led to such circumstances, and that the woman may repeat the same behavior in the future.

Of course, prevention is important; at Jikei Hospital, they perform counseling services for pregnancy, childbirth, and child rearing that are achieving results. In Germany, repeated consultations lead to parents deciding not to use a babyklappe in most cases, and even if they do use it, approximately half of them reclaim their children within eight weeks [1,6,18]. Stating that drop-offs at baby hatches should be prevented and that social childcare mechanisms should be appropriately used is only natural. However, the above objections can be refuted.

First, with regard to individual parents neglecting their child raising obligations, society as a whole has a responsibility to protect and raise children. It is irresponsible to criticize only the parents who drop off a child; only by working together can we prevent such parents from abandoning their children [19]. This is “our” problem, the collective responsibility of we who have made society the way it is. Rather than ascribing pregnancy and childbirth to the individual responsibility of women, we should work on solving the problem with the attitude that the entire society will support them by enhancement of counseling frameworks for pregnancy and childbirth, livelihood support for single mothers [18].

Second, regarding the argument that baby hatches encourage child abandonment, we question whether child abandonment is so easily triggered. Might such thinking be based on a prejudiced view—that parents who would go through a pregnancy and childbirth requiring concealment from others are good-for-nothings and lack proper human nature, and therefore easily dispose of their own child? Yet, no parent gladly disposes of their own child. Parents who have left their babies unattended in a dangerous place to die have simply become isolated from their families and society, incapable of obtaining adequate information to make appropriate judgments, and mentally trapped [19]. Furthermore, the individual responsibility of women regarding pregnancy, childbirth, and contraception is still emphasized, and governments thus far have not devised adequate measures for the socially vulnerable. There are, in fact, women in poverty who are unaware of government support systems [18].

Third, dropping a child off at a baby hatch is not simply child neglect. The parents drop off their child at a safe place hoping that he or she survives; hence, he or she is not a so-called deserted child. We may thus assert that the act clearly differs from a dangerous one such as leaving a child unattended on the street. Jikei Hospital President Taiji Hasuda, who established the “Stork’s Cradle” in Kumamoto, writes on the hospital website that “deserting a child may cause it to lose its life. But in the act of dropping off one’s child at a safe place, is there not the mother’s fervent desire to save her own child?” [20] A German doctor points out that most of the children dropped at *Babyklappes* are relatively healthy and the purpose of dropping off a child is to protect its life and that mothers who drop off their children are definitely not bad mothers [6].

Fourth, as mentioned previously, parents in a difficult situation may feel institutional or psychological resistance in approaching public child welfare organizations. Fifth, it is pointed out that the social childcare center is pressed by rapidly increasing consultations concerning child abuse and understaffing is the severe state, and among those who had actually consulted with it, there existed some parents who let their child die by abuse or finally chose to drop a baby off at a baby hatch. As a matter of facts, despite the involvement of the social childcare center in 45 cases, seven children died in 2010 [1,6,21].

Finally, we argue that education to publicize social childcare systems and prevent unwanted pregnancies is difficult. Although education is important, the information is not always conveyed effectively to those who need it. Even if publicized on a website, there will always be a certain proportion of people who do not or cannot access the information. No matter how much we teach appropriate coping methods, some people will not use them. There are limits to educational and awareness-raising activities.

#### Some people abuse the baby hatch, using it for selfish reasons

According to a 2012 report, the primary reasons for dropping off a child at the Cradle were poverty, objections of parents (that is, of grandparents), unmarried, and a parent’s mental disorder, among others. These are arguably unavoidable and acceptable reasons [1]. We may speculate that there were many pressing situations in which parents thought, “if we don’t drop off the child now, both mother and child will be unable to live properly, and it will be a life-and-death matter for the baby,” and after an agonizing decision, left the newborn child in a baby hatch as a last resort.

Meanwhile, in the Kumamoto prefecture 2009 report, doubts such as “one can glimpse a distorted sense of belonging” were cast upon reasons such as “the family register will be stained” [6]. The 2012 report also states that there were multiple cases in which there was no absolute need to leave the child at the Cradle, such as cases in which a child was dropped off because the parents could not quickly find a childcare facility so they could work, or would not be able to raise the child because they were going to study overseas [1]. The acceptability of a reason cannot be judged so simply without considering the details of each individual case. Yet, compared to the first group of reasons, the latter group appears to be less acceptable; parents could not complain if they were declared to be prioritizing only their own happiness. In addition, there was an extremely unfortunate case of a child being used for a financially-motivated crime in which an underage guardian dropped a child off at the Cradle, embezzled property that the child had inherited, and was subsequently arrested [1,22]. In some cases, infants (13.6%) and toddlers (7.4%) are dropped off instead of newborns [1]. The first child dropped off at Jikei Hospital’s “Stork’s Cradle” was a three-year-old toddler [23].

Obviously, abuse must be prevented. The purpose of the baby hatch is to accommodate newborn infants as a last resort, so it should be clearly stated as such and be thoroughly publicized to prevent improper use. In the case of Jikei Hospital, they have been operating the “SOS baby and mother telephone counseling office” on a 24-h basis from December 2006, before operation of the Cradle began, so that babies are not casually dropped off. In practice, they have been able to significantly prevent improper use [1,6]. Here, we can again refute the position that establishment of baby hatches is bad because of abuse.

First, we need to determine whether baby hatches are inappropriate just because some people abuse them. Whatever the system, unfortunately, there are always some people that will abuse it. Even if baby hatches have been used for simplistic reasons, if this only applies to some people and most parents had pressing reasons, then would not the system be acceptable? Criticism should be directed at the abusers, rather than casting doubt upon the entire baby hatch system just because some use them for selfish reasons.

Second, are we capable of readily determining the simplicity behind the reason for another’s behavior? Indeed, from a third-person perspective, we think “how could they drop off a child for such a reason!” However, even if an act is foolish when examined calmly, the person concerned may have been in an extremely serious and desperate psychological state at that point in time [24]. When considering someone else’s problems, we can all easily become great philosophers and judges. Although an objective judgment from a third-person perspective is important, the subjective psychology and feelings of the concerned parties are also important.

Third, Atsushi Yamada responds to the criticism “perhaps they will drop children off casually,” as follows: “Even so, what matters is that lives are saved. Although the ‘simplicity’ of those that drop off a child and the ‘deterioration of ethical values’ are pointed out, we should attend more to the reality of a society in which child-rearing is difficult. Women are always the ones that struggle. That men are not held responsible is also a problem. Each individual citizen must become aware of the reality that many babies are dying” [6].

#### Baby hatches cannot save babies’ lives

In Germany, the incidence of neonatal death and abandonment has not decreased since introduction of the babyklappe [25]. While the babyklappe may help troubled women, some people believe they do not help the children [15]. However, survey results from one region of one country for a defined time period can hardly be called reliable evidence. We could also claim that the baby hatch counteracted what would otherwise have been an increase in child abandonment [24]. In addition, there are the data which indicate that the number of abuse death of children has slightly been increasing in Japan, and it is suggested unwanted pregnancy could lead to it. In 2010, there were 51 cases of abuse death and most of sacrificed children were under 1 years old [1,6]. The increase of the abuse death lets us concern about the increase of the future abandoned child, and it may be said that the necessity of baby hatch rises more.

While some claim that the system saves a significant number of newborn lives as a fact, others argue that demand has been created out of nothing [5,17]. What would have happened to the 81 children that were dropped off during a five-year period at the baby hatch of Jikei Hospital? Although we can speculate, the truth is unknowable: the claim that they would have been abandoned on the streets or killed is as valid as the claim that they would have been raised safely by their parents. Unless collaborative long-term social experiments are carried out in many regions of multiple countries to produce solid data, we will only see a clash of beliefs and assertions devoid of evidence.

#### The rights of one parent can be ignored if the other surrenders a child without his or her consent

There is concern that baby hatches may be used by unscrupulous fathers, step-fathers, relatives, or even controllers of prostitutes to pressure mothers to dispose of an unwanted baby. Therefore, the big question is if baby hatches are upholding women's rights and if the mother consents to her baby being placed there [3]. Needless to say, there is no excusing a male partner or other family member who, ignoring the mother’s intentions, deserts the child of a mother who wishes to raise it; this is a cruel and criminal act that must never occur. However, such situations will arise regardless of whether or not baby hatches exist. They do not occur because baby hatches exist. If there are no baby hatches, the likelihood that these children will be left on the street unattended or killed will increase. A clear distinction should be made between outcomes that occur only because baby hatches exist and those that occur regardless of their existence.

#### The baby hatch puts a baby in medical jeopardy

Childbirth must take place under medical supervision [1]. However, giving birth at home alone is likely to occur regardless of whether or not baby hatches exist. Another argument is that the safety of the child is not guaranteed when it arrives from far away immediately after birth; however, this objection supports the establishment of baby hatches in various places. In response to the suggestion that safety of the child prior to drop-off is not secured (e.g., plane travel immediately after birth), Hospital President Taiji Hasuda stated, “There is a limit to accommodating children at Jikei Hospital alone. I would like similar facilities to be built at several places around the country” [26]. Those who assert that baby hatches should be abolished based on a problem that will potentially be resolved by expanding access to baby hatches are those who are likely to reject baby hatches out of hand. The establishment of baby hatches or other anonymous delivery systems is likely to reduce dangerous childbirths.

It was reported that there were 99 urgent consultation and management performed by the Jikei Hospital about pregnancy and delivery and about half came from the residents in Kumamoto prefecture in the first three years after the establishment of the “Stork’s Cradle.” But the actual use of the “Cradle” by the consultation clients who lived in Kumamoto has not been confirmed [6]. This result suggests that local direct and upmost measures involving a family as well as parents are effective for the parents to find alternatives other than the use of the baby hatch. Therefore, it is necessary to establish more local institutions such as the “Stork’s Cradle” which offers a baby hatch as well as preventive consultation and support system nationwide.

#### The baby hatch has no clear legal basis

Finally, there is opposition to baby hatches because they are in a legal gray area. In Australia, a woman’s right to anonymous delivery does not exist, and the right to know one’s parents is considered a basic children’s right. However, anonymous delivery and the use of baby hatches is recognized as legal if a condition of poverty that exposes the lives, as well as the mental and physical health, of the woman and child to unavoidable danger is confirmed [18]. The legal position of babyklappes remains unclear in Germany [9]. In Japan, in February 2007, the state indicated its position is that baby hatches “are not outright illegal.” As a result, on April 5 of the same year, Kumamoto City determined that “there are no reasonable grounds for not allowing modifications in the medical law,” and approved changes that allowed establishment of the “Storks’ Cradle” [1].

Numerous rebuttals can be made in response to the objection that the legality of baby hatches is unclear. Ambiguous legality and illegality differ. That which is ethically correct can exist, even if it is illegal. Laws are not for approving every action in daily life, but to prohibit actions that must not be performed in order to maintain social order by punishing violators. That which is legal in one country may be illegal in another, or that which is legal in one era may be illegal in another. Therefore, the ethical propriety of a matter cannot be determined solely on the basis of legal judgments, or whether or not there are laws in place.

## Summary

### Should we maintain baby hatches in our society?

Should baby hatches continue to exist? Which is preferable: a world with baby hatches or a world without them? Furthermore, should we build more than we currently have? We agree with the establishment of the “Stork’s Cradle” at Jikei Hospital in Kumamoto City. A list of benefits of baby hatches are presented below. There are numerous possible refutations for the major objections mentioned above. Attempts to open and operate baby hatches spontaneously, either privately or as an organization, should not be criticized; rather, they should be applauded. If a facility is initiated with good intentions, the maximum possible efforts are made to protect the interests, rights, and safety of children at the facility of concern, and there is no clear evidence of harm to the mother, child, or society, then we think that the facility should be supported. To prevent the baby hatches from losing their function, the ultimate decision of whether to drop off the child in anonymity should be left to the parents.

A list of benefits of baby hatches:

Baby hatches can give children the right to life and they can be saved.

Baby hatches can protect vulnerable pregnant women who are mentally and economically desperate.

Baby hatches can uphold parent’s right to anonymity and both stigmatization and discrimination against them can be avoided.

Baby hatches can have the societal function of serving as a last resort for desperate women and/or parents.

Baby hatches can provide an alternative of social childcare organizations with only limited function.

Baby hatch can serve as the emergency shelter role temporarily which accepts the baby of the mother and/or parents who need time to think.

Baby hatches can fulfill social responsibility to protect and raise children by realizing a principle of solidarity.

Baby hatches can offer pregnant women and/or parents an opportunity to drop off their child at a safe place hoping that he or she survives.

Baby hatches can serve as important local direct and upmost measures for women and/or parents in trouble, by offering 24-hour preventive consultation and support system.

Baby hatches can be a symbol embodying human compassion which is a pure and natural feeling that it is unbearable and impossible to remain indifferent in the face of another’s misfortune.

Baby hatches can urge society as a whole to deliberate current situation in which child-rearing is difficult.

Is it “an activity to support the safety and independence of the mother and child by preventing child abandonment and child murder,” or “a device that encourages easy child abandonment” [5]? In fact, no one knows. Although there are arguments for and against baby hatches, clear and solid evidence for these arguments is unavailable at the present time. However, human beings die very easily. If we are to err, then it is better to make attempts that seem beneficial to life even in the slightest degree. We consider baby hatches to be an embodiment of human compassion which is a pure and natural feeling that it is unbearable and impossible to remain indifferent in the face of another’s misfortune [27].

Not all human beings are capable of communicating well with others, being self-assertive, or asking for help in a timely manner. If they were, then tens of thousands of people per country would not commit suicide each year, nor would people kill themselves out of desperation induced by bullying. Not all human beings are careful and responsible either. Some are weak-willed. There are thoughtless people as well as cowardly people. There are complete egoists. There are also cunning men, and women who have children despite knowing that their partners are such men. Some people are not blessed with partners or relatives. Some grandparents will be unsympathetic. Furthermore, in this world, there are no perfect systems or life-saving facilities. We should consider the continuation of baby hatches with such realities in mind. Baby hatches are necessary in our present society. Though it is best not to be used, it is a place of socially essential emergency refuge for babies and parents.

## Competing interests

Atsushi Asai and Hiroko Ishimoto declare that we have no competing interests.

## Authors’ contributions

Both of AA and HI have equally made substantial contributions to conception, design, acquisition and interpretation of references; have been involved in drafting the manuscript and revising it critically for important intellectual content; and have given final approval of the version to be published.

## Pre-publication history

The pre-publication history for this paper can be accessed here:

http://www.biomedcentral.com/1472-6939/14/9/prepub
